# Hierarchical Morphing Control of an Ultra-Lightweight Electro-Actuated Polymer Telescope with Thin-Film Actuators

**DOI:** 10.3390/mi14101855

**Published:** 2023-09-28

**Authors:** Kainan Wang, Yuxuan Gong, Yian Yu, André Preumont

**Affiliations:** 1 School of Remote Sensing and Information Engineering, Hubei Luojia Laboratory, Wuhan University, Wuhan 430072, China; 2 Department of Control Engineering and System Analysis, Université Libre de Bruxelles (ULB), CP. 165-55, 50 Av. F.D. Roosevelt, B-1050 Brussels, Belgium

**Keywords:** ultra-lightweight telescope, ferroelectric thin film, non-contact actuation, active optics, hierarchical control

## Abstract

This paper explores advanced shape control techniques for ultra-lightweight electro-actuated polymers with composite ferroelectric thin films. It begins with an overview of PVDF-TrFE film actuators used in the development of thin-shell composites, emphasizing the need to overcome constraints related to the electrode size for successful scalability. Strain generation in thin-film actuators is investigated, including conventional electrode-based methods and non-contact electron flux excitation. Numerical studies incorporate experimentally calibrated ferroelectric parameters, modeling non-contact actuation with an equivalent circuit representation. The potential distribution generated by electron flux injection highlights its potential for reducing print-through actuation issues. Additionally, the paper outlines a vision for the future of large thin-shell reflectors by integrating the discussed methods for charging ferroelectric polymer films. A hierarchical control strategy is proposed, combining macro- and micro-scale techniques to rectify shape errors in lightweight reflectors. These strategies offer the potential to enhance precision and performance in future spaceborne observation systems, benefiting space exploration and communication technologies.

## 1. Introduction

**Figure 1:**
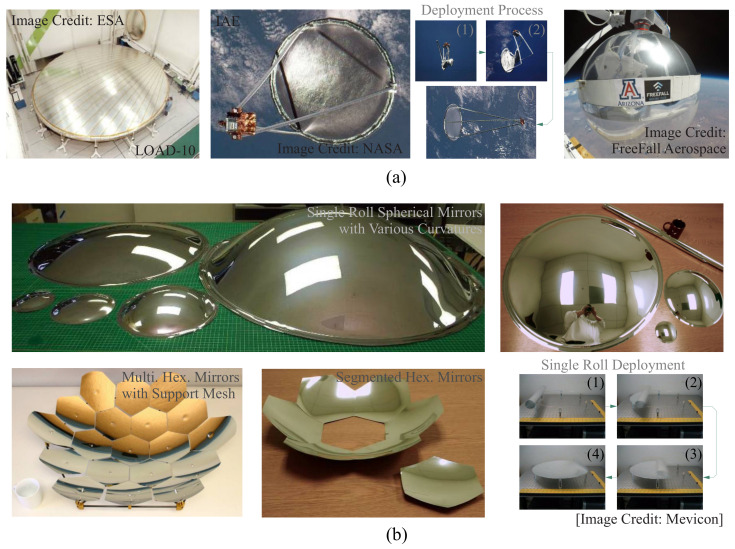
Literature survey of the reflectors made of polymer materials. (**a**) Inflatable balloon and antennas for ground tests and used in space, including the LOAD-series reflector model (image credit: ESA, Paris, France) [6], IAE deployment tests in space (image credit: NASA, Washington, DC, USA), spherical inflatable membrane carried by small satellites (image credit: FreeFall Aerospace, Inc., Tucson, AZ, USA) [7,8]; (**b**) form-stiffened thin-shell prototypes with various forms of apertures and the deployment test (image credit: Mevicon, Inc., Mountain View, CA, USA) [4,5].

The use of polymer reflectors in space observation is gaining attention within the aerospace community due to their ultralow areal density and stowability. These qualities make them a promising option for future space missions, despite the challenges of maintaining their accuracy in harsh space environments. There are two broad classes of space reflectors where polymer materials can potentially be used. The first one is the inflatable balloon configuration, in this approach, polymer films are used to create inflatable balloons that form the reflective surface [1]. These balloons can be inflated to create a rigid structure in space, and this concept is verified in early space experiments, e.g., NASA’s inflatable antenna experiment (IAE) launched in 1996 [2]. The structural rigidity is maintained by pressure stiffening, wherein the pressure inside the balloon keeps the surface taut and accurate; moreover, a chemical rigidization process is also in development with ultraviolet (UV) radiation in space to reduce the weight of the carry-on gas for long-term missions [3]. The second configuration is the form-stiffened thin shell, which involves using polymer materials to create thin-shell reflectors with inherent stiffness generated by the surface curvature [4]; this is consistent with the need for designing conic surfaces for the reflection of electromagnetic waves. These reflectors can be stowed during launch and space transportation in a compact form and then released in space using their own elastic strain energy; a flexible arrangement of the aperture (e.g., hexagonally segmented or monolithic) is also possible [5] for this concept. This method provides an alternative to inflatable balloons for maintaining the shape of the reflector. Figure 1 presents several examples of reflectors made of polymer materials with those concepts.

Deploying polymer reflectors for precision engineering in space is filled with formidable challenges due to the extreme conditions of the space environment. Several factors, including volatile thermal fluctuations, erosive effects caused by atomic oxygen, and deformations resulting from stress releases, pose significant threats to the success of space observation missions. To address these challenges, various classes of polymers can be considered for constructing the reflective surface, such as polyimide (PI) [4,9,10], polyethylene terephthalate (PET) [1,11], and nitrocellulose [12,13]. Here, we emphasize the key material properties that are critical for effective reflector designs.

- *Thermal considerations:* The space environment is subject to wide temperature variations, leading to significant deformations in polymer-based reflector substrates and resulting shape errors. Therefore, the selection of materials with minimal coefficients of thermal expansion (CTE) is of utmost importance. Ideally, a polymer material with zero CTE is highly desirable, as exemplified by $Novastrat^{®}$ (developed by ManTech, Herndon, VA, USA), which boasts an exceptionally low CTE of approximately 0.4ppm/K [14,15]. On the other hand, thermal stability is also crucial, considering the viscoelastic properties, including creep, which significantly influence the behaviors of thin-shell mirrors constructed from polymers during moving phases, such as unfolding deployment. Maintaining long-term dimensional stability necessitates continuous monitoring of surface figures, often requiring the implementation of feedback control systems.
- *Surface quality:* The reflectivity of polymer thin-shell mirrors is significantly impacted by the surface roughness following the coating process. The attainment of a smooth micro-scale surface on the thin mirror substrate is imperative for the effective implementation of macro-scale active control. This underscores the critical necessity of selecting substrate materials endowed with exceptional mechanical properties suitable for the thermoplastic forming process. Typically, reported values for surface roughness in the references cited [4,5,9,12,13] consistently fall below $R_{a}<20$ nm. However, when considering the shape error control of the wavefront within a macro-scale clear pupil, the root mean square (RMS) error $\sigma_{W}$ of the surface figure should conform to specific standards. For imaging applications, achieving a Strehl ratio of 0.8 necessitates [16]
  (1) $$\sigma_{W}<\frac{\lambda }{28}$$
  where λ represents the detection wavelength. For non-imaging applications, e.g., incoherent LiDAR, a relaxed criterion can be applied with a factor of 5, i.e., $\sigma_{W}<\lambda$/6 should be fulfilled [5].
- *Space compatibility:* Particular attention is dedicated to assessing the material’s resistance to space-related threats, including exposure to solar radiation, particle radiation, and erosive factors. These environmental challenges have the potential to compromise the long-term viability of the polymer material and, consequently, the service life of the space reflector. It is worth noting that NASA has conducted an extensive series of experiments as part of the Materials International Space Station Experiment (MISSE) program to tackle these formidable challenges [17].

While there are currently limited space applications, a brief literature review [1,5] provides insights into the best achievable surface quality documented thus far for the ground-test measurements, exhibiting an RMS error of approximately 20 μm when the pupil is scaled to a diameter of 0.1 m. According to Equation (1), for such a level of surface accuracy, a practical wavelength is estimated to be around λ≈560μm. This wavelength falls within the transition region between the microwave and far-infrared electromagnetic radiation. In addition to the passively operated reflectors, a new generation of reflectors equipped with active shape control is emerging, inspired by the concept of active optics (AO), and offering a promising solution that can attain the requisite surface accuracy needed for the utilization of shorter wavelengths. This innovation is exemplified by references such as [13,18].

**Figure 2:**
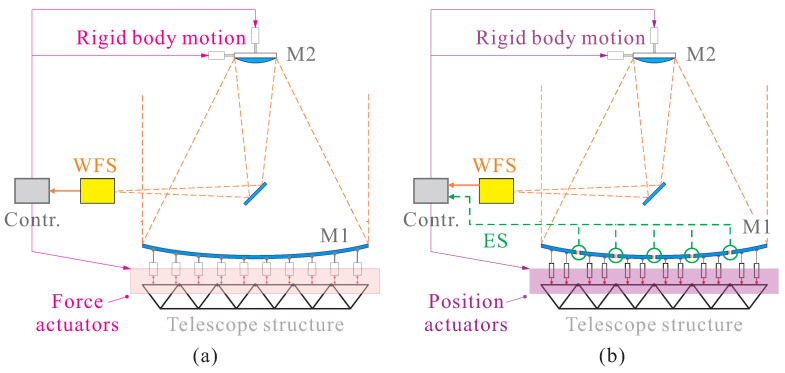
Fundamental principles and typical implementations of active optics (AO) in large telescopes. (**a**) Feedback AO implementation with force actuators on a ground telescope with a monolithic primary; (**b**) feedback AO implementation with position actuators on segmented mirrors. M1: primary mirror, M2: secondary mirror, WFS: wavefront sensor, ES: edge sensor.

The concept of active optics was initially implemented in giant terrestrial telescopes, where it involved stabilizing the telescope structure to mitigate the effects of various factors stemming from the ground environment. Its primary objective is to correct shape errors in large primary mirrors induced by ground-based factors, such as aerodynamic disturbances (wind), microscale seismic activity, gravitational sag, and thermal gradients. The fundamental principles and typical implementations of ground-based active optics (AO) are depicted in Figure 2a,b, with those equipped with actuators generating out-of-plane forces in a feedback loop, either through force control on a monolithic mirror, as seen in examples like ESO’s VLT telescopes [19], or by employing position controls for segmented mirrors, e.g., the Keck telescopes [20], where the segmented mirrors are co-phased to function as a monolithic unit using edge sensors. Wavefront sensors are incorporated to monitor mirror deformations, transmitting these signals to the controller. The controller then generates actuation commands within specific bandwidths.

In contrast to ground-based applications that often employ substantial actuators for morphing the control of large primary mirrors, in-plane strain control presents a viable alternative, showcasing significant potential due to its compact nature. In addition, a high stroke-deformable mirror (DM) might also be involved to correct the residual error of the incoming wavefront within a certain field-of-view (FoV) [21]. To actuate the thin-shell substrate effectively, it is essential to consider an electro-active polymer with ferroelectric properties. The most common options include poly(vinylidene fluoride (PVDF) or its copolymers designed to enhance performance, such as poly(vinylidene fluoride-co-hexafluoropropylene) (PVDF-HFP) or poly(vinylidene fluoride-trifluoroethylene) (PVDF-TrFE). These materials serve as the foundation for strain actuators. The morphing control is made by attaching active thin films onto the coated thin-shell substrate, alongside an array of electrodes; as a common method, by applying a specific pattern of electric potentials, these strain actuators generate bending moments and extension (membrane) forces along the electrode contour [21,22]. This precise manipulation allows for the controlled deformation of the mirror, enabling it to achieve the desired shape with accuracy and efficiency; practical examples of active polymer reflectors can be found in [18,23].

In this paper, we begin with a brief review of a technology demonstrator developed at Université Libre de Bruxelles (ULB) and manufactured in collaboration with Materia Nova. In Section 2, we delve into various technical aspects aimed at increasing the diameter of polymer reflectors, thereby necessitating the exploration of non-contact methods for generating strain with high-resolution morphing capabilities. In Section 3, we initially explore the conventional method of strain actuation in the transverse mode, which involves charging a pair of parallel electrodes. This method also capitalizes on the nonlinear ferroelectric behavior of thin film, with critical parameters of film material properties obtained through experimental calibration. Subsequently, we embark on a novel approach to excite thin piezoelectric films using electron flux; we conduct numerical investigations with an equivalent circuit model and undertake a comparative study to evaluate their morphing behavior on a curved thin-shell substrate. Finally, in Section 4, we present a hierarchical morphing strategy and validate it through numerical simulations on a large aperture, ultra-lightweight structure actuated by the active polymer film.

## 2. Development of Thin-Shell Composite with Film Actuators

**Figure 3:**
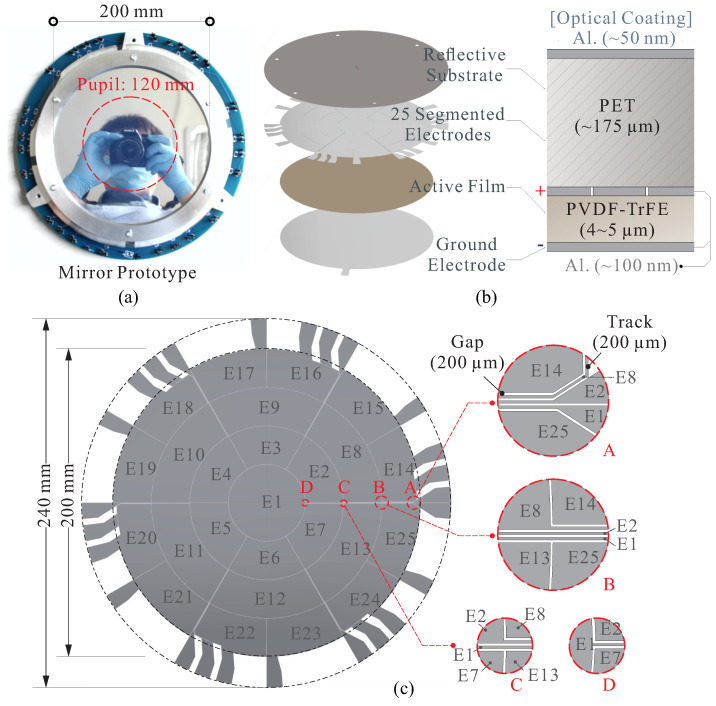
Thin shell composite with film actuators. (**a**) The mirror demonstrator; (**b**) the layers of the composite with a PET substrate and a thin film of PVDF-TrFE; (**c**) patterned electrodes [18].

In this section, we begin by providing a concise overview of the technology demonstrator developed at Université Libre de Bruxelles (ULB), which was manufactured in collaboration with Materia Nova, and supported by the ESA project [18,23]. The mirror prototype is depicted in Figure 3a, featuring a diameter of D=0.2 m, and the clear pupil designated for morphing tests is $D_{P}=0.12$ m. The thin composite mirror is secured by a pair of ring supports, comprising layers consisting of a 175 μm-thick coated PET substrate and a thin film of ferroelectric PVDF-TrFE, 4 to 5 μm in thickness. The patterned electrodes (positive) are positioned in between, with the ground electrode located on the exterior, as shown in Figure 3b.

Figure 3c depicts the patterned aluminum electrodes that were deposited onto a transparent polymer sheet with a radius of curvature (RoC) of $R_{C}=2.5$ m. The metal electrode deposition is carried out using a magnetron sputtering technique and subsequently obtained through masked lithography. The electrode pattern is designed in a keystone layout, consisting of 25 independent electrodes with tracks guiding the electrical connections outside the clear pupil [18]. In order to evaluate the performance of shape control, a relative morphing test is conducted. To form certain optical modes (Zernike modes), a shape-fitting error of $\sigma_{W}<300$ nm in RMS for each mode can be achieved. This outcome is particularly encouraging as it demonstrates the feasibility of utilizing thin-shell mirrors to operate in an infrared range superior to those offered by passive alternatives.

Achieving accurate formation of these mode shapes necessitates a meticulous arrangement of the actuator array. It has been extensively documented that the design of the electrodes should adhere to the criterion $D_{E}<\sqrt{R_{C}t_{S}}$ [24], where $D_{E}$ represents the dimension of the electrode, and $t_{S}$ denotes the thickness of the substrate layer.

The limitation imposed by the electrode size presents significant challenges, especially when designing a large-diameter reflector with active control. Consider the example of the primary mirror of the James Webb Space Telescope (JWST), which has a diameter of D=6.5 m and a RoC of $R_{C}\approx 15.9$ m. If we intend to use a thin-shell with a thickness of 200 μm, the required number of electrodes, denoted as *N*, must satisfy the condition $N\geq D^{2}/R_{C}t_{S}\approx 13286$. Meeting this requirement can be highly demanding when it comes to the design and implementation of the high-voltage unit, as it necessitates a tremendous number of channels for independent control. Additionally, it becomes practically impossible to create a well-structured pattern of electrodes, especially when dealing with complex layouts of tracking routes. This reality underscores the need for alternative and more efficient methods of stimulating the active film with high resolution and precise control maintaining an acceptable level of complexity and feasibility.

## 3. Strain Generated by Thin-Film Actuators

In this section, our exploration begins with an examination of the conventional method for transverse strain actuation, which entails the application of an electric field between a pair of parallel electrodes. We specifically employ a nonlinear ferroelectric model to incorporate electrostrictive strain, an approach highly suitable for thin-film actuators; an experimental calibration is conducted to determine those material parameters. As the refinement of the active reflector’s shape directly relies on the number of electrodes, there is a natural inclination to utilize a greater number of actuators. However, doing this might present significant technical challenges due to the intricate nature of the high-voltage electrical unit required for charging films. In this paper, we propose an alternative non-contact actuation model. Here, we initiate our study by examining the potential on the film surface by injecting the electron flux using an equivalent circuit model. To enhance the flexibility of precise strain control, we introduce a back pressure voltage for a compound control. This model is initially explored in one dimension, and subsequently, we extend it to investigate spatially distributed potential on the film. This expansion allows us to thoroughly investigate morphing behavior and make comparisons with conventional actuation methods.

### 3.1. Strain Actuation by Charging a Pair of Parallel Electrodes

The conventional method for inducing actuation strain in a thin ferroelectric film is to employ a pair of parallel electrodes (refer to Figure 3b) and apply a controllable voltage $V_{a}$ to these electrodes. The electric field is oriented perpendicular to the electrodes (labeled as 3), resulting in strain within the film occurring horizontally (labeled as 1). The in-plane strain $S_{1}$ generated by electric excitation along the poling direction is given by $S_{1}=Q_{31}P_{3}^{2}$ for dielectric materials, where $P_{3}$ is the polarization in the out-of-plane direction. In the case of a ferroelectric film, the polarization can be expressed as $P_{3}=P_{S}+\varepsilon_{0}\varepsilon_{r}E_{3}$ under a first-order assumption, where $P_{S}$ represents the level of spontaneous polarization saturation, and $\varepsilon_{0}\varepsilon_{r}E_{3}$ is polarization induced by the external electric field $E_{3}$ [23], the actuated strain can be written as

(2) $$S_{1}=Q_{31}{\left(P_{S}+\varepsilon_{0}\varepsilon_{r}E_{3}\right)}^{2}=Q_{31}P_{S}^{2}+2\varepsilon_{0}\varepsilon_{r}Q_{31}P_{S}E_{3}+Q_{31}\left(\varepsilon_{0}\varepsilon_{r}\right)E_{3}^{2}$$

where the first term, $Q_{31}P_{S}^{2}$, represents the poling strain, which remains permanent with a polarized sample; the second term, $2\varepsilon_{0}\varepsilon_{r}Q_{31}P_{S}E_{3}$, represents the piezoelectric strain, where the nominal piezoelectric constant can be defined by $d_{31}=\partial S_{1}/\partial E_{3}=2\varepsilon_{0}\varepsilon_{r}Q_{31}P_{S}$ with no external bias field applied; the third term, ${\left(\varepsilon_{0}\varepsilon_{r}\right)}^{2}Q_{31}E_{3}^{2}$, is the electrostrictive strain, which becomes dominant when the active film is thin. Notice that the in-plane electrostrictive coefficient $Q_{31}=-\nu Q_{33}$, and $Q_{33}$ is considered constant, serving as a material property for dielectrics.

Two methods for depositing the active film onto the substrate are commonly employed. The first method is spin coating, which involves the centrifugal spreading of a viscous liquid drop containing the solvent of PVDF-TrFE. The second method is spray coating, where a high-pressure spray flow is directed onto the substrate. The uniformity of film thickness is often constrained when using spin coating due to the viscosity of the solvent. In contrast, the spray coating technique overcomes this limitation, allowing for the production of larger-sized samples.

Following the deposition process, it becomes essential to subject the composite to an elevated temperature for annealing. This annealing process transforms the paraelectric phase of PVDF-TrFE into the ferroelectric phase, paving the way for subsequent poling procedures. The ferroelectric parameters of the film, associated with Equation (2), are experimentally measured and summarized in Table 1, together with the data obtained in [18]. In particular, samples of spray-coated PVDF-TrFE subjected to varying annealing temperatures were examined. Notably, the dielectric constant ($\varepsilon_{r}$) values were found to be lower for the spray-coated samples compared to the spin-coated ones. This discrepancy in the dielectric constant seems to reduce the piezoelectric actuation capability of the spray-coated samples.

According to the classical Stoney formula [25], the surface curvature $\kappa_{S}$ of a morphing thin mirror substrate can be estimated by

(3) $$\kappa_{S}=6S_{1}\frac{Y_{F}}{Y_{S}}\frac{1-\nu_{S}}{1-\nu_{F}}\frac{t_{F}}{t_{S}^{2}}$$

where *Y*, ν, and *t* represent the Young modulus, Poisson ratio, and layer thickness, respectively. The subscripts *S* and *F* indicate the substrate and thin film, respectively. We can substitute Equation (2) for Equation (3), which results in the surface curvatures by various driving sources, presented as follows:

(4) $$\kappa_{S,P}=\frac{12\varepsilon_{0}\varepsilon_{r}Q_{31}P_{S}Y_{F}\left(1-\nu_{S}\right)}{Y_{S}t_{S}^{2}\left(1-\nu_{F}\right)}V_{a}$$

(5) $$\kappa_{S,E}=\frac{6Q_{31}{\left(\varepsilon_{0}\varepsilon_{r}\right)}^{2}Y_{F}\left(1-\nu_{S}\right)}{Y_{S}t_{S}^{2}\left(1-\nu_{F}\right)}V_{a}^{2}/t_{F}$$

**Figure 4:**
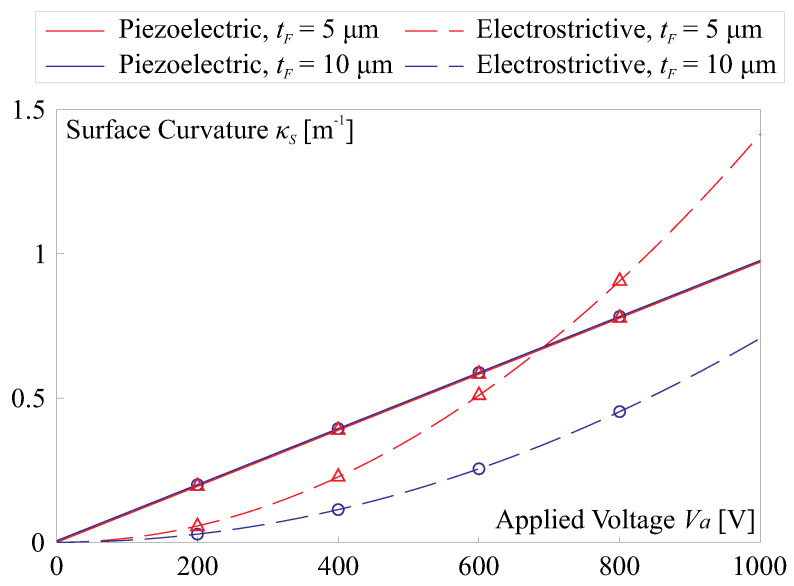
Surface curvatures induced by the piezoelectric and electrostrictive actuation with various thicknesses of the active film ($t_{F}=5\;\mu$m and 10 μm); the results are computed using Equations (4) and (5), respectively. This numerical example uses experimentally calibrated data, as follows: $Y_{F}=2.5$ GPa, $Y_{S}=5.6$ GPa, $\nu_{F}=0.34$, $\nu_{S}=0.38$, $t_{S}=200\;\mu$m.

Figure 4 presents the computed results of surface curvatures induced by the piezoelectric and electrostrictive actuations, concerning the applied voltage $V_{a}$, where various thicknesses of the active film are investigated. It is worth noting that, although the piezoelectric strain (the second term in Equation (2)) is directly proportional to the electric field $E_{3}$, the resulting curvature $\kappa_{S,P}$ it generates behaves linearly with the voltage $V_{a}$, regardless of the film thickness $t_{F}$. This behavior arises as the equivalent moment $M_{E}$ generated by a unimorph film is proportional to $S_{1}t_{F}$ [21,22]. However, differences become apparent when the film extension is driven by electrostrictive strain. According to Equation (5), the resulting curvature is related quadratically to the voltage and inversely proportional to the film thickness. This suggests that a thinner active layer leads to a larger morphing amplitude.

### 3.2. Numerical Study on Strain Excitation with Electron Flux

Another method of exciting the ferroelectric film involves injecting an electron flux directly into the film, which is typically accomplished using an electron gun. This approach is designed to achieve exceptionally high resolution for correcting shape errors across a wide range of spatial frequencies. The initial stages of this research were undertaken at the University of Kentucky, involving extensive experimental investigations into the strain response of piezoelectric materials under electron flux. These investigations encompassed both polymers [26] and ceramics [27,28]. The method capitalizes on secondary electron emission by regulating the electron flux intensity on one side of the film while simultaneously controlling the potential difference, in conjunction with a back pressure voltage, on the opposite side. It is noteworthy that, in this scenario, the metal coating for electrode purposes is applied solely to the side with the back pressure voltage. From a theoretical modeling perspective, many approaches have been conducted qualitatively, relying on techniques, such as the diagrammatic approach [27] and theoretical deductions utilizing principles from quantum mechanics [28].

**Figure 5:**
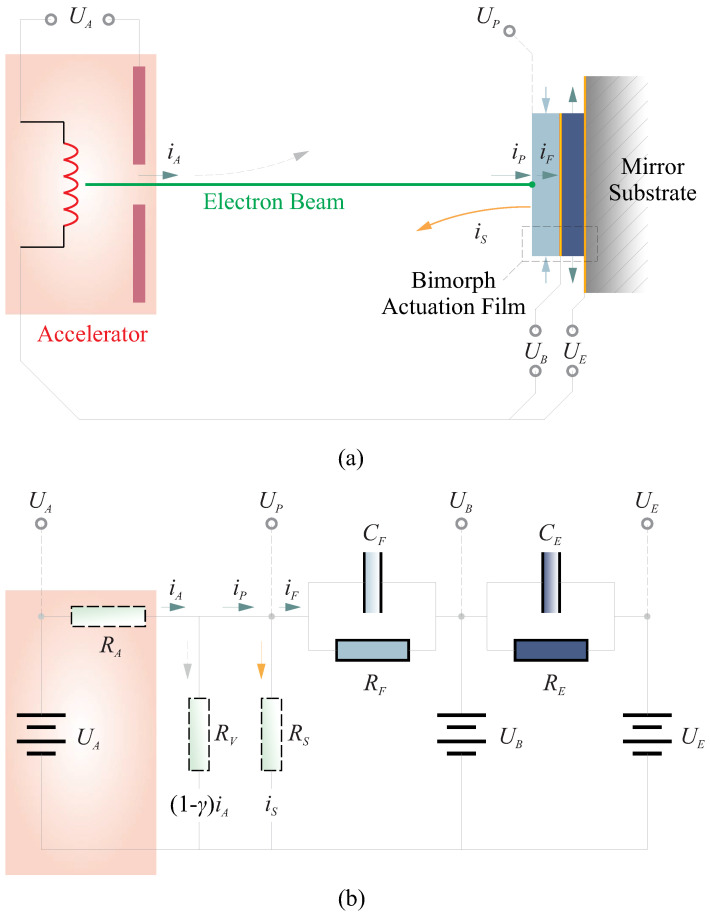
Modeling the strain excitation of the thin ferroelectric film with the electron flux. (**a**) The schematic representation of the strain actuation process; (**b**) the equivalent circuit model.

In this paper, we developed a model based on an equivalent circuit, rooted in the principles of charge conservation along the electron transmission pathway; although there is a lack of experimental verification, this study aims to propose a reasonable phenomenological model that awaits future experimental investigations for critical comparison. The implementation of the strain actuation process is depicted in Figure 5a, while the corresponding equivalent circuit is presented in Figure 5b (adapted from [29]). In this model, the electron gun is simulated as an accelerator consisting of a pair of cathode and anode, with an applied potential of $U_{A}$ to accelerate the electrons as they exit. In our design, the ferroelectric films are arranged in a bimorph configuration, as will be utilized in Section 4. The first layer serves to control micro-scale shape errors using the electron flux, with the strain regulation achieved through the surface potential $U_{P}$ and the backside potential $U_{B}$. The second layer addresses macro-scale shape corrections, following the actuation approach outlined in Section 3.1, generating strain in relation to the potential difference $U_{B}-U_{E}$ (both $U_{B}$ and $U_{E}$ being controllable).

According to the Child–Langmuir law [30], when electrons are emitted from the cathode of an electron gun and accelerated by a voltage of $U_{A}$, the maximum possible current density $j_{A}$ (measured in A/m${}^{2}$) under a space-limited charge distribution can be described as:

(6) $$j_{A}=\frac{4\varepsilon_{0}}{9}\sqrt{\frac{2e}{m}}\frac{U_{A}^{3/2}}{x_{A}^{2}}$$

Here, *e* and *m* represent the charge and rest mass of an electron, respectively. The electron accelerator is represented by a pair of flat plates separated by a distance of $x_{A}$ and subjected to a potential difference of $U_{A}$. It is important to note that the classical Child–Langmuir law assumes that the kinetic energy of the electrons at the cathode is $E_{0}=0$, thus neglecting the initial velocities of the thermally emitted electrons. As a result, it is often considered an underestimation of the current density. The total output current at the electron gun can be calculated as $i_{A}=j_{A}A_{S}$, where $A_{S}$ represents the spot area.

**Figure 6:**
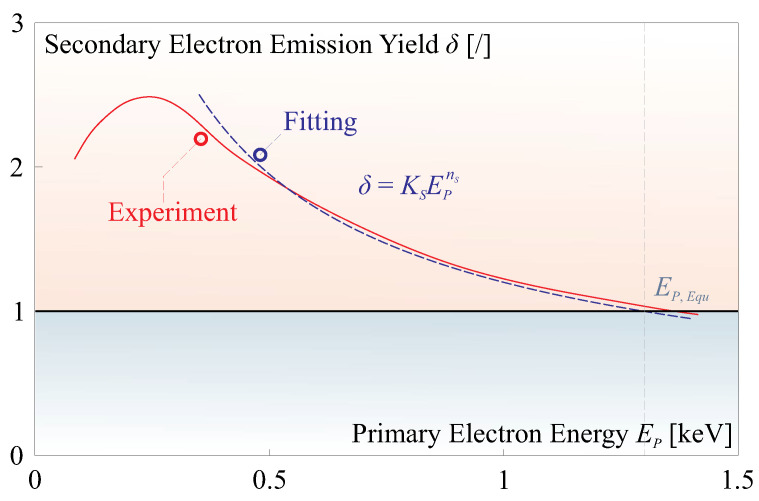
Literature reviews on the $\delta -E_{P}$ curve of the PVDF sample [31].

The electron flux that reaches the surface of the ferroelectric film carries a current denoted as $i_{P}$. However, there is a loss of charge during this transmission, quantified by a factor of γ, i.e., $i_{P}=\gamma i_{A}$. The total current at the input port of the film can be divided into two components: (1) the current that passes through the film, represented as $i_{F}$, the film has a thickness of $t_{F}$; (2) the returning current, denoted as $i_{S}$, which is a consequence of secondary electron emission. This secondary emission is influenced by the energy of the electrons impacting the surface $E_{P}$. Estimations for $i_{S}$ can be performed using experimentally calibrated secondary electron yield relationships, often characterized by the parameter $\delta =|i_{S}/i_{P}|$. These relationships are typically presented in the form of a $\delta -E_{P}$ curve. In the case of a thin film of PVDF, numerical fitting of this $\delta -E_{P}$ curve can be found in [31]. It can be mathematically expressed as $\delta =K_{S}E_{P}^{n_{S}}$ and shown in Figure 6.

The passing through the current can, therefore, be written by

(7) $$i_{F}=i_{P}-i_{S}=\gamma \left(K_{S}E_{P}^{n_{S}}-1\right)\frac{4\varepsilon_{0}}{9}\sqrt{\frac{2e}{m}}\frac{U_{A}^{3/2}}{x_{A}^{2}}A_{S}$$

On the other hand, the thin film can be also simulated using an equivalent capacitor with a capacitance of $C_{F}$ in parallel with a resistance of $R_{F}$, within the scanned spot size $A_{S}$, $C_{F}=\varepsilon_{r}\varepsilon_{0}A_{S}/t_{F}$ ($\varepsilon_{r}$ is the relative permittivity) and $R_{F}=t_{F}/\kappa A_{S}$ (κ is the electrical conductivity), then the current $i_{F}$ can be written by

(8) $$i_{F}=C_{F}\frac{d(U_{P}-U_{B})}{dt}+\frac{U_{P}-U_{B}}{R_{F}}$$

where $U_{B}$ is the back pressure voltage (controlled variable), and $U_{P}$ is the potential for solving.

**Figure 7:**
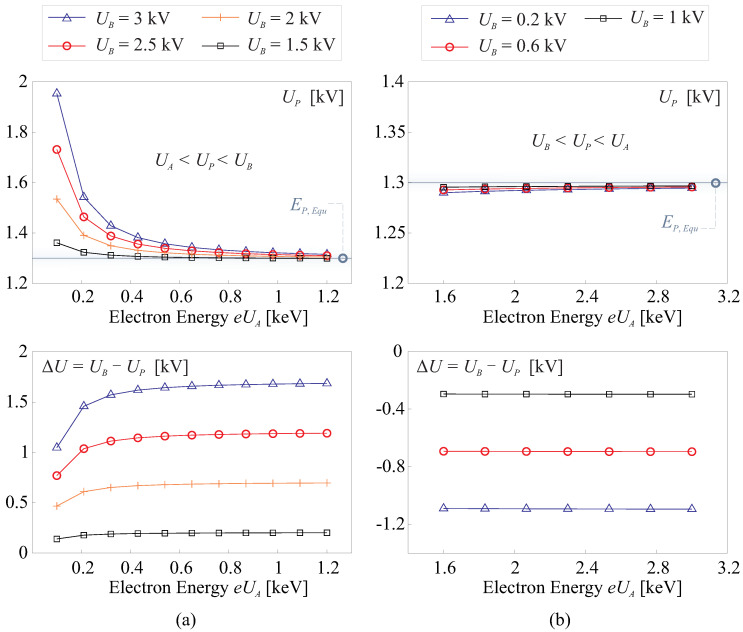
Quasi-static solutions of $U_{P}$, the top plots show the solutions of $U_{P}$ and the bottom plots given the potential difference $\Delta U=U_{B}-U_{P}$. (**a**) $U_{A}<U_{P}<U_{B}$; (**b**) $U_{B}<U_{P}<U_{A}$.

By combining Equations (7) and (8) while applying a quasi-static assumption to the circuit model illustrated in Figure 5b (omitting the derivative term in Equation (8)), we can derive a solution for $U_{P}$, which represents the potential at the input port of the film. This solution typically falls into two distinct scenarios, as visually depicted in Figure 7, the top plots show the solutions of $U_{P}$ and the bottom plots show the potential difference $\Delta U=U_{B}-U_{P}$. Following parameters are used for this simulation: $A_{S}=1\times 10^{-4}$ m${}^{2}$, $t_{F}=5\;\mu$m, $x_{A}=5\times 10^{-2}$ m, γ=0.9, $K_{S}=151$, $n_{S}=-0.7$.

The first scenario occurs when $U_{A}<U_{P}<U_{B}$, as illustrated in Figure 7a. Typically, the back pressure voltage $U_{B}$ is considerably higher than the potential of the electron gun $U_{A}$. To implement control and adjust the potential difference $\Delta U=U_{B}-U_{P}$, one can generally tune the energy of the electron gun $eU_{A}$, while maintaining a base supply of $U_{B}$. Maintaining $U_{B}$ quasi-statically constant serves to simplify the control process and is also necessary since $U_{B}$ is also utilized to regulate the potential difference $\Delta U_{M}=U_{B}-U_{E}$ between the electrodes of the macro-patches. The second scenario, $U_{B}<U_{P}<U_{A}$, illustrated in Figure 7b, presents a solution where $U_{P}$ is nearly constant and equals the equilibrium solution of $\delta =K_{S}E_{P}^{n_{S}}=1$. In this case, controlling the potential difference becomes straightforward, as it involves independently tuning $U_{B}$. This approach is similar to the application described in [27,28].

**Figure 8:**
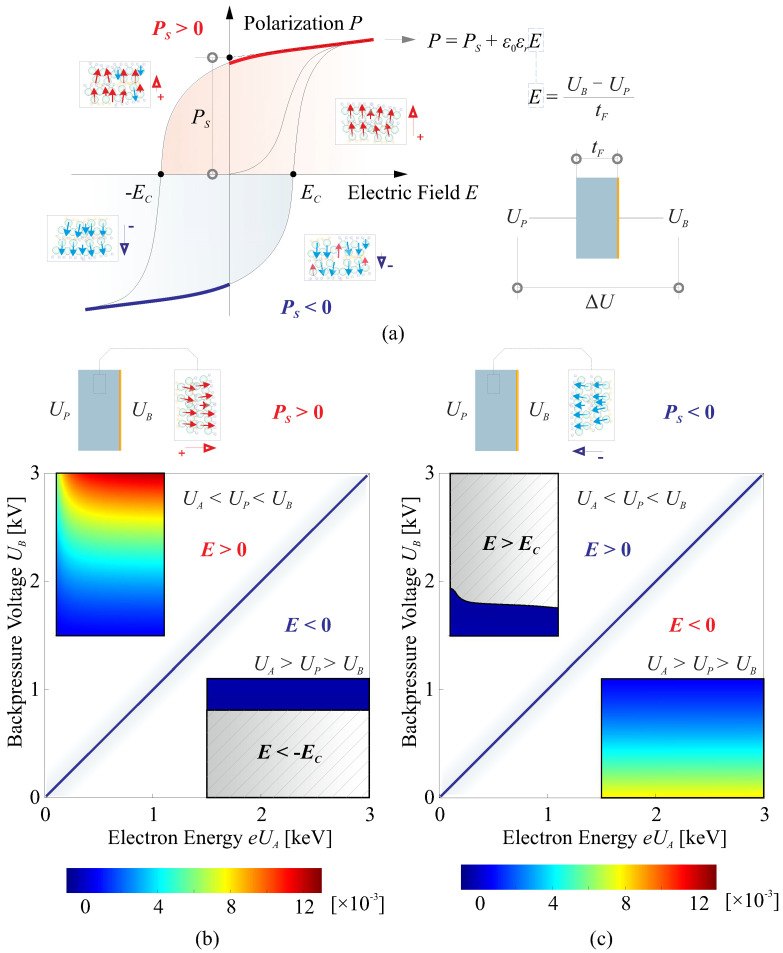
Quasi-static computation of the generated strain follows Equation (2). (**a**) The P−E relationship of a ferroelectric material; (**b**) the generated strain resulting from a combined adjustment of both emission energy $eU_{A}$ and backside voltage $U_{B}$ within the applicable range, the polarization direction exhibit a positive dipole orientation towards the backside; (**c**) the generated strain resulting from a combined adjustment of both emission energy $eU_{A}$ and backside voltage $U_{B}$ within the applicable range, the polarization direction exhibit a positive dipole orientation toward the polymer surface encountering the electron flux.

The computation of the generated quasi-static strain follows Equation (2), with an electric field input of $E=\left(U_{B}-U_{P}\right)/t_{F}$ at the excited node within this one-dimensional model. The resulting strain also depends on the direction of the polarization *P*, as depicted in Figure 8a. When the applied electric field *E* aligns with the direction of the dipoles, the film undergoes stretching ($S_{1}>0$ due to $d_{31}>0$), while if the field opposes the dipole direction, the film experiences compression ($S_{1}<0$). It is worth noting that when the magnitude of the negative field is sufficiently large, i.e., $E<-E_{C}$, where $E_{C}$ represents the coercive field, the polarization will be reversed.

Figure 8b,c offer insights into the generated strain resulting from a combined adjustment of both emission energy $eU_{A}$ and backside voltage $U_{B}$, taking into account different directions of poling within the applicable range. When the goal is to achieve a specific desired strain by modifying the energy $eU_{A}$, it corresponds to the scenario depicted in the top left of Figure 8b. In this case, the polarization direction should exhibit a positive dipole orientation towards the backside. It is worth noting that the poling process differs from the case presented in Figure 3. Due to the absence of coating electrodes on the surface of the thin polymer film, a plasma poling method is required. This poling method has been explored in relevant research [32].

Another critical aspect of testing involves evaluating the dynamic responses of the strain generated in response to voltage changes, the derivative term $C_{F}d\left(U_{P}-U_{B}\right)/dt$ in Equation (8) should be taken into account. This aspect is particularly significant when considering the application of using a small number of electron guns to control a large thin-shell reflector (as will be discussed in Section 4).

**Figure 9:**
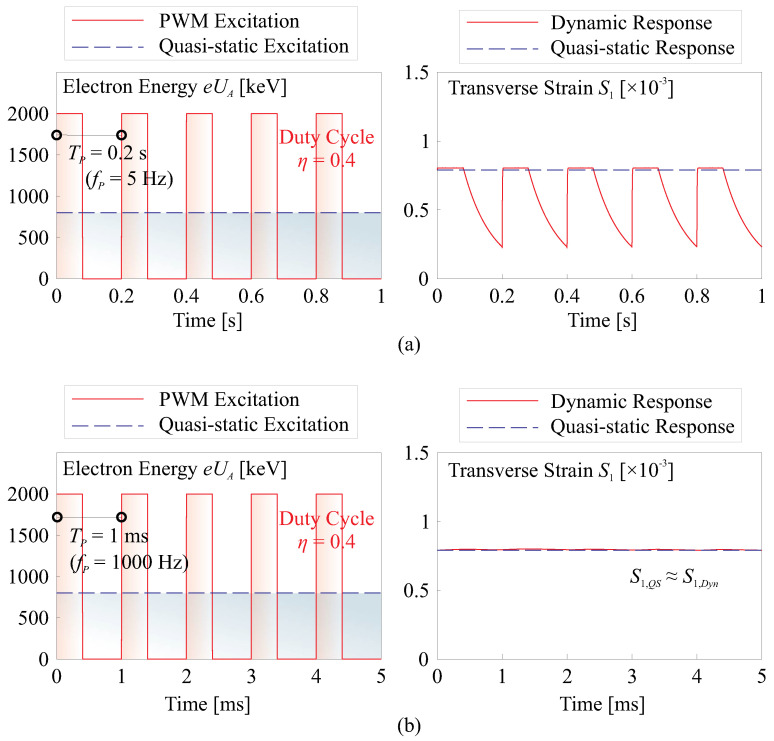
Dynamic excitation of the morphing film by applying a PWM signal of $eU_{A}$; input signals are on the left, and the resulting strains for both dynamic and quasi-static excitations are compared on the right. (**a**) $f_{P}=5$ Hz; (**b**) $f_{P}=1000$ Hz.

In this paper, a pulse width modulation (PWM) excitation signal is employed to adjust the input energy $eU_{A}$ and generate strain levels equivalent to those achieved with quasi-static excitation. Numerical examples illustrating this dynamic excitation signal are presented in Figure 9. The dynamic excitation signal utilizes a PWM with the same energy as its quasi-static counterpart, and the excitation frequency $f_{P}$ should be determined to align with the specific requirements of the application. A general requirement for ensuring equivalence with the quasi-static scenario can be expressed as

(9) $$f_{P}\gg \frac{1}{R_{F}C_{F}}=\frac{\kappa }{\varepsilon_{r}\varepsilon_{0}}$$

This condition helps ensure that the dynamic excitation effectively bridges the gap with the quasi-static equivalence, as high-frequency excitation signals can pass through the capacitor. In the numerical example provided, the Runge–Kutta method is employed to solve the equation. The input parameters of $\kappa =1\times 10^{-9}$ S/m${}^{2}$ and $\varepsilon_{r}=12.5$ yield a frequency value of $\kappa /\varepsilon_{r}\varepsilon_{0}\approx 9$ Hz.

### 3.3. Comparative Study on Morphing Capability on a Thin-Shell Curved Reflector

Up to this point, our investigation into the electron flux excitation has been primarily focused on a one-dimensional model, centered around the excited node without considering the spatial distribution of the potential. While we have used the spot size factor $A_{S}$ in the model, it has had minimal impact, mainly relating to the magnitude of the pass-through current. In this section, we delve into the morphing behavior of the composite thin-shell under the excitation of non-uniform potential. To begin, we briefly review the morphing mechanics of charging a pair of parallel electrodes. As reported in [21,22], isotropic piezoelectric film actuators, when driven by a constant field *E* within the electrode contour, experience equivalent loads acting on the actuator’s edge. These loads consist of an extension force per unit length, $F_{E}=\left[d_{31}Y_{F}/t_{F}\left(1-\nu_{F}\right)\right]E$, and a surrounding bending moment, $M_{E}=\left[d_{31}\left(t_{S}+t_{F}\right)Y_{F}/2t_{F}\left(1-\nu_{F}\right)\right]E$. However, if the surface is not flat, which is often the case in optical design, additional pressure must be applied, quantified as $P_{E}=2F_{E}\kappa_{M}$. Here, $\kappa_{M}$ represents the invariant mean curvature on the surface, as discussed in [33]. Another form of equivalence is related to morphing induced by thermal strain with an equivalent temperature, which in nature represents self-equilibrium strain actuation.

**Figure 10:**
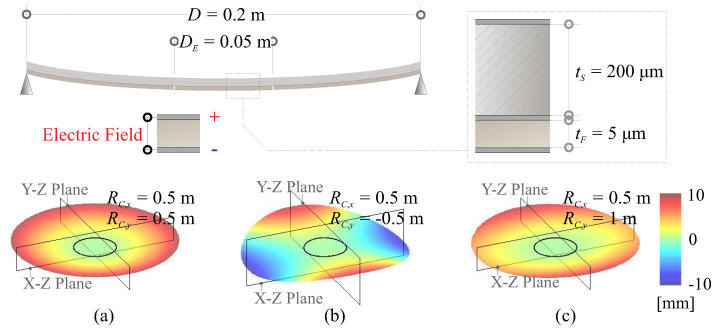
Tested examples for numerical verification of strain morphing examination, (**a**) $R_{C,x}=0.5$ m, $R_{C,y}=0.5$ m; (**b**) $R_{C,x}=0.5$ m, $R_{C,y}=-0.5$ m; (**c**) $R_{C,x}=0.5$ m, $R_{C,y}=1$ m.

Figure 10 presents tested examples for numerical verification of strain morphing examination for a unimorph composite mirror. In these tests, a uniform potential is applied to the electrode. The composite reflector features a PET substrate of 200 μm and a thin actuation film of 5 μm. The mirror has a diameter of D=200 mm and is supported isostatically on the edge. The electrode size is $D_{E}=50$ mm.

**Figure 11:**
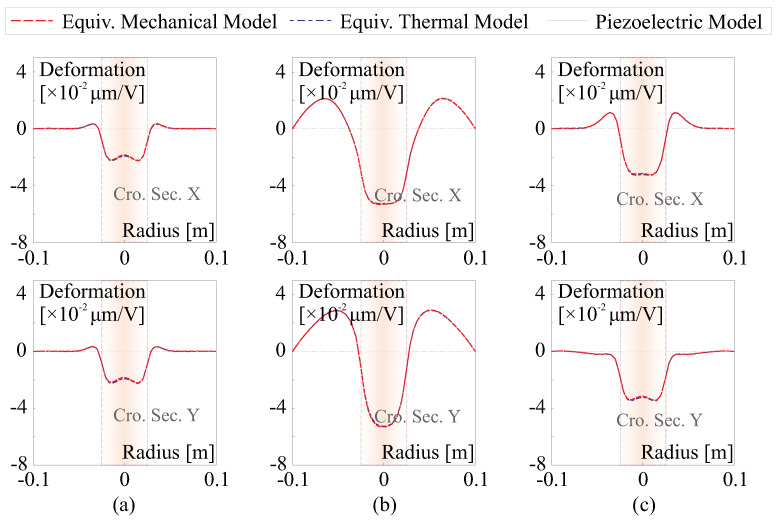
Tested examples for numerical verification of strain morphing examination, (**a**) $R_{C,x}=0.5$ m, $R_{C,y}=0.5$ m; (**b**) $R_{C,x}=0.5$ m, $R_{C,y}=-0.5$ m; (**c**) $R_{C,x}=0.5$ m, $R_{C,y}=1$ m.

Three cases of biconic mirrors are examined, demonstrating perfect agreement between the strain actuation model and its mechanical and thermal equivalences, as shown in Figure 11. There is a notable behavior that can deteriorate the morphing performance in highly curved thin shells, leading to the print-through of the actuator. The quantitative criteria proposed in [21] suggest that the print-through phenomenon will begin to appear if $D_{E}/\sqrt{R_{C}t_{S}}\geq 2.76$ and become significant if $D_{E}/\sqrt{R_{C}t_{S}}\geq 5.9$ (assuming $t_{S}\gg t_{F}$); e.g., in Figure 11a, where $D_{E}/\sqrt{R_{C}t_{S}}\approx 5$, we observe that the cross-section resembles the contour of the electrode superimposed with the fluctuated wavy shape on the edge.

However, when considering an electron flux impacting the side of the dielectric polymer, it is important to note that the resulting potential may not have a guaranteed uniform distribution. Presently, there is no established theoretical formulation for the spatial distribution of the potential acting on the thin film due to its complex nature. Nevertheless, numerous experimental investigations have been conducted on this issue, e.g., as documented in [34]. In this paper, the potential difference caused by the electron flux injection is represented as a function $\Delta V\left(r\right)=U_{B}-U_{P}$ in radial coordinate *r*, with $D_{S}$ quantifying the influencing area. There are certain (reasonable) constraints assumed on the mathematical formulation of this profile function, including:

- V(r) is an even function, meaning that V(r)=V(−r), and at the origin (r=0), the maximum value of *V* is $V\left(r\right)=V_{a}$.
- V(r) is a monotonically decreasing function within the range of r∈[0,+∞], specifically, dV/dr<0 for r>0.
- V(r) is a convex function at the origin, i.e., $d^{2}V/dr^{2}{|}_{r=0}<0$.

Weak constraints may also be applied, such as the potential being negligibly small at the border of the influencing area (i.e., $V(D_{S}/2)\approx 0$) and the profile function being concave (i.e., $d^{2}V/dr^{2}{|}_{r=\pm D_{S}/2}>0$). In the current study, two possible profile functions are examined: (1) the trigonometric function, $V\left(r\right)=V_{a}\left[1+\cos\left(2\pi r/D_{S}\right)\right]/2$; (2) the Gaussian function, $V\left(r\right)=V_{a}e^{-4.6{\left(2r/D_{S}\right)}^{2}}$. The first profile function meets all the necessary requirements outlined in the previous assumptions. In the second one, the factor of −4.6 indicates that there is approximately 1% of the remaining magnitude at the edge, specifically at $r=\pm D_{S}/2$.

**Figure 12:**
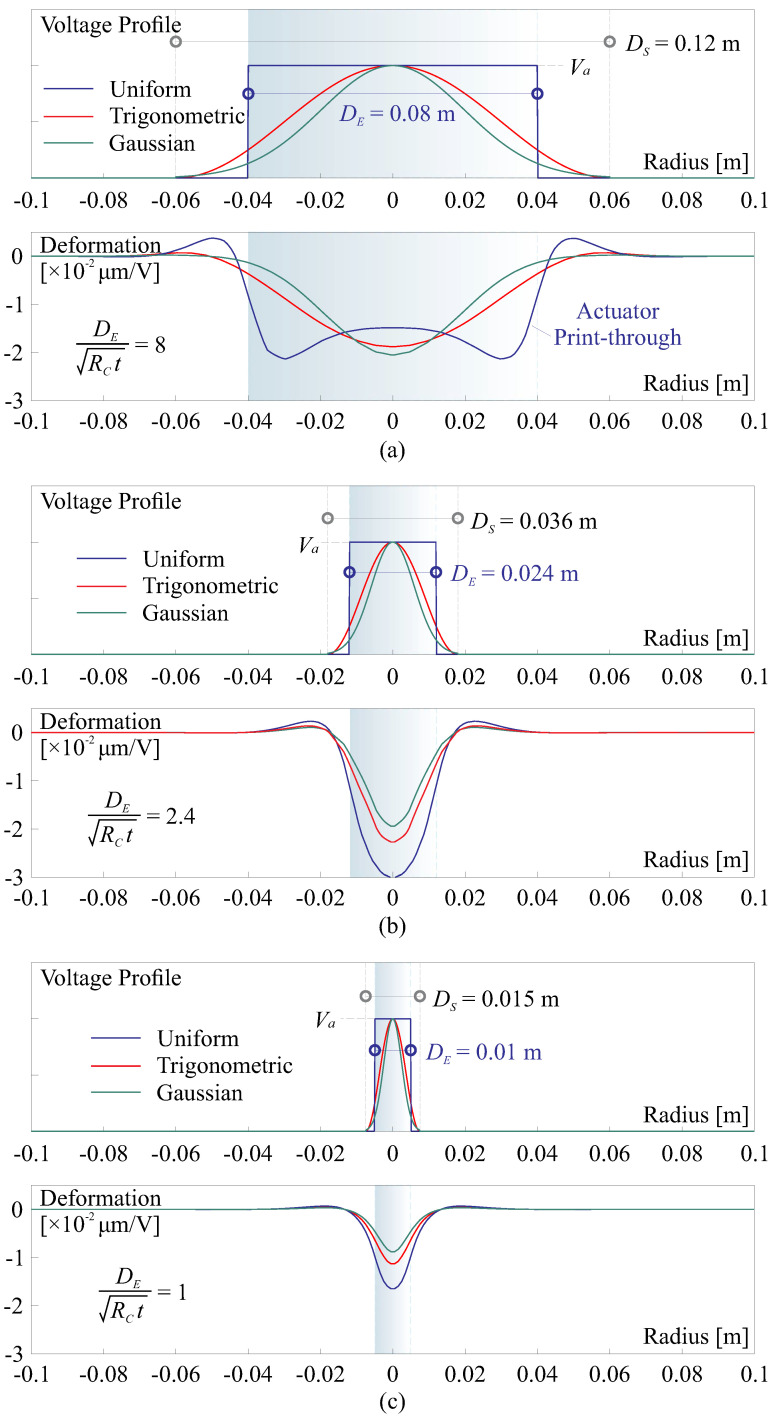
Comparison of morphing behaviors in cross-sections among excitation methods including the application of voltages by two assumed profile functions and a uniform magnitude, various electrode sizes of $D_{E}$ are tested with $D_{S}=1.5D_{E}$. (**a**) $D_{E}=8\sqrt{R_{C}t_{S}}$; (**b**) $D_{E}=2.4\sqrt{R_{C}t_{S}}$; (**c**) $D_{E}=\sqrt{R_{C}t_{S}}$.

Figure 12 provides numerical examples illustrating the application of these two assumed profile functions with various influencing diameters ($D_{S}$) and compares the resulting deformation to that induced by a uniform field $E=V_{a}/t_{F}$ with the various electrode sizes of $D_{E}$, the potential is applied on a thin-shell with the layers of Figure 10 and the RoC of $R_{C}=0.5$ m. For a general comparison, we can assume a fixed relation of $D_{S}=\eta D_{E}$, where $\pi D_{S}^{2}/4$ is regarded as an equivalence of the spot area $A_{S}$, it is common that η>1 indicating the spreading electron causes extra potential on the boundary; in these numerical tests, it is set by η=1.5.

In Figure 12a, where $D_{E}=8\sqrt{R_{C}t_{S}}$, we observe distinct advantages for the electron flux excitation method compared to using a uniform voltage. This electron flux method eliminates the morphing shape of the actuator’s print-through and slightly increases the morphing amplitude. There is a slight difference in the morphing behavior for the potential distribution of the trigonometric and Gaussian functions. Figure 12b,c depict two cases, where $D_{E}<2.76\sqrt{R_{C}t_{S}}$, resulting in the disappearance of the actuator’s print-through in the electrode charge driving mode. For both examples, the morphing amplitudes are reduced when using the electron flux to deform the thin shell. The reduction is more pronounced for the Gaussian function approximated profile than for the trigonometric one, as it contains less energy (quantified by the volume within the range of $r<D_{S}/2$ of the profile function). It is important to note that while the trigonometric and Gaussian functions serve as phenomenological models, and the value of η still needs to be experimentally calibrated, these observations can be applied in general situations. Additionally, it is important to note that the deformation profile created by the electron injection method also adheres to the constraints set for V(r). This aspect is particularly significant because the mechanical responses can be viewed as a magnification of a unit pulse with a Gaussian profile, which simplifies the model construction of the interaction matrix.

**Figure 13:**
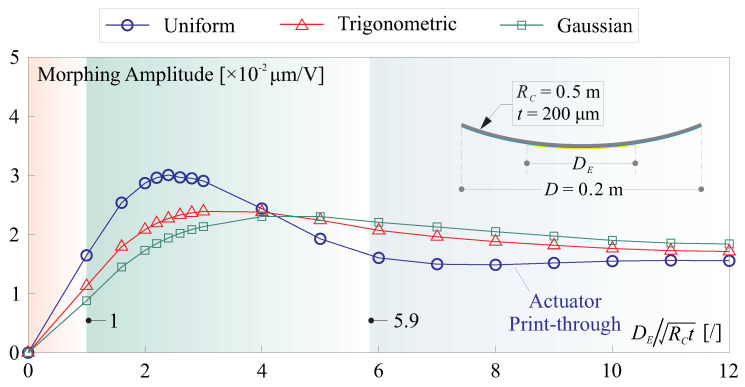
Change in morphing amplitude with respect to the actuation size quantified by $D_{E}$ (and corresponding $D_{S}$) for various excitation methods.

Figure 13 presents the change in morphing amplitude with respect to actuation size quantified by $D_{E}$ (and corresponding $D_{S}$), exhibiting a similar trend as observed in [21]. The results consistently show that the morphing amplitudes are limited, regardless of the driving method used. This limitation is normal, as it is primarily caused by the intrinsic stiffness introduced by the shape curvature. Specifically, excitation by the electron flux, which eliminates the problem of the print-through, is expected to correct high-frequency errors with low magnitudes (complied with the nature of a surface as described in [33,35]), as discussed in Section 4.

## 4. Hierarchical Control of a Large Thin-Shell Surface: A Future Perspective

In this section, we offer a future perspective on controlling large-sized thin-shell reflectors by integrating the methods discussed earlier for charging thin films of ferroelectric polymers. While several practical challenges may currently restrict the immediate implementation of such active reflectors, this research lays the groundwork for their potential use in space observation and signal communication in the future.

**Figure 14:**
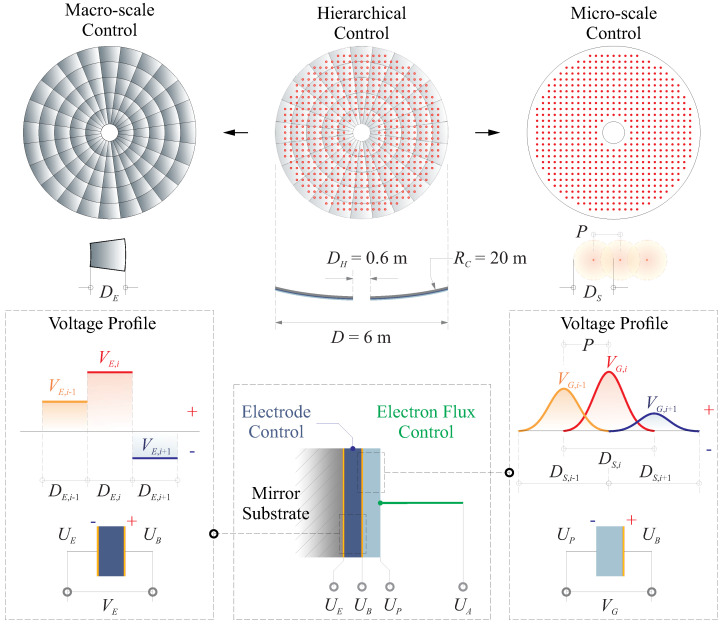
Illustrative diagram the schematic of the proposed compound control method. The macro-scale shape control uses an array of patterned actuators equipped with independent channels depicted in Section 3.1 and the micro-scale shape control involves modulating the electron flux with controllable injecting energies investigated in Section 3.2.

Figure 14 illustrates the schematic of this compound control method. It involves correcting shape errors of low-order modes (with low spatial frequencies) in thin-shell reflectors using an array of patterned actuators equipped with independent channels for voltage amplification. In the traditional approach, the number of channels required is determined by the equivalent size of the electrode relative to the geometric factor $\sqrt{R_{C}t_{S}}$. However, this approach is not technically feasible for systems with a large number of controlled degrees of freedom (DoF). Instead, to address the difficulties in correcting high spatial frequency shape errors, the morphing method considers exciting the active thin film with an electron flux with modulated power, while also adjusting the potential on the backside. This approach is aimed at compensating errors that may arise from factors like the manufacturing process, internal stress release, or residual errors from previous control stages.

In this paper, we present the simplest implementation of the hierarchical control algorithm discussed earlier, assuming perfect optical wavefront sensing. Our setup features an active spherical reflector with a diameter of D=6 m (with a central obstruction of 10%) and a radius of curvature $R_{C}=20$ m. The design of the polymer material layers follows the numerical example depicted in Figure 10. In the first step of the hierarchical control process, we employ a macro-scale control strategy utilizing a Keystone pattern of electrodes. These electrodes have a dimension of $D_{E}/\sqrt{R_{C}t_{S}}\approx 8.54$, where $D_{E}$ represents the length of the sector electrodes evenly distributed along the radial direction. This array design provides us with 120 independent control degrees of freedom (DoFs), as illustrated in Figure 14. The second step of our shape refinement process involves using a tunable electron flux to correct micro-scale errors in the shape. In this method, we create a typical actuator pattern in Cartesian coordinates using a scanning mechanism. These actuation spots are separated by a pitch dimension denoted as *P*. Currently, without critical experimental verification, we approximate the resulting morphing shape with a Gaussian profile having an influencing size of $D_{S}$. We assume that this dimension is linearly related to *P* by a factor of $D_{S}=\chi P$, where χ>1 due to interactions among adjacent actuator nodes. The charging architectures of the thin film are illustrated at the bottom of Figure 14.

**Figure 15:**
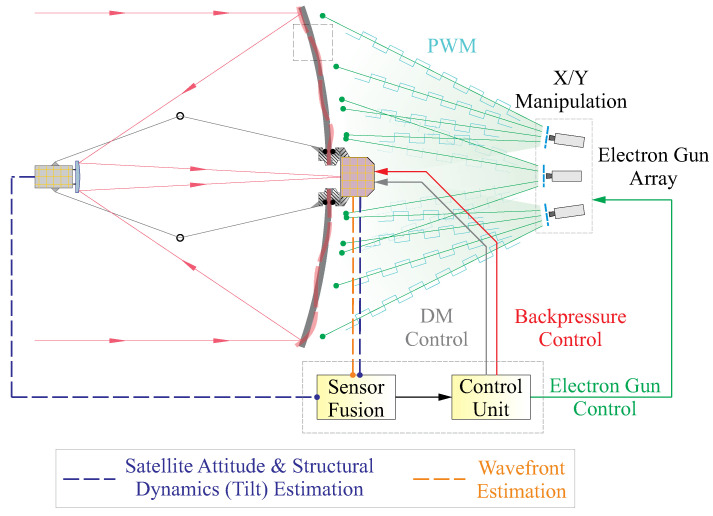
Ultimate vision of a future lightweight space telescope with a sensor fusion technique.

Furthermore, as presented in Figure 15, future space telescopes are expected (ultimate vision) to compensate for wavefront errors within a sufficient frequency bandwidth and various spatial patterns by coordinating subunits of active optics components in an integrated spaceborne observation system. The control scheme is based on sensor fusion techniques, which combine wavefront error data received by optical sensors and structural status information reconstructed by synthesizing the inertial attitude sensor signals on the satellite. This approach will enable the space telescope to achieve sharp imaging within a certain field of view (FoV) and proposed in future studies.

**Figure 16:**
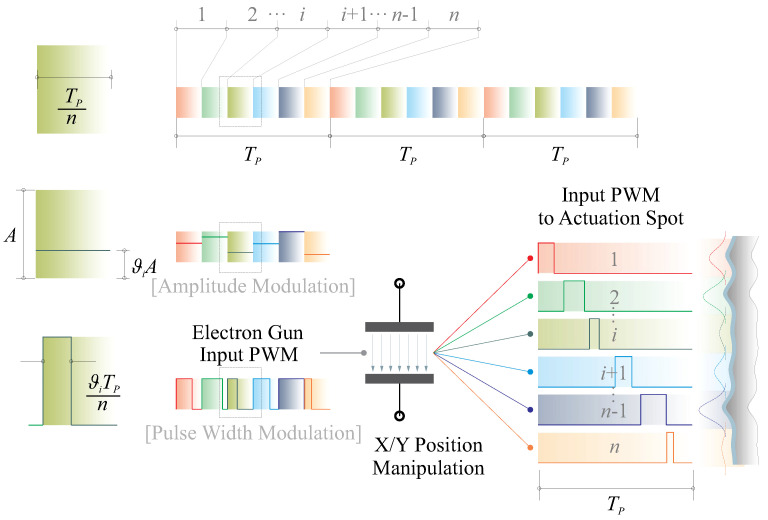
Baseline design of assigning the emitted power of the electron flux, one actuator (the electron gun) is assumed to control a number of actuation nodes (denoted by *n*), generating interacting morphing spots to correct micro-scale errors.

In Figure 16, we propose a baseline design that involves assigning the emitted power of the electron flux. In this approach, one actuator (the electron gun) is assumed to control a number of actuation nodes (denoted by *n*), generating interacting morphing spots to correct micro-scale errors. This design is inspired by the simulated work shown in Figure 9. In one period $T_{P}$ of the dynamic signal, the actuation time slots are divided into *n* segments. For the $i^{th}$ node, this division should be further multiplied by a factor of $\vartheta_{i}$. Therefore, the overall duty cycle for the $i^{th}$ actuation spot is $\vartheta_{i}/n$. The core issue of this control method is to set an appropriate value of the frequency $f_{P}=1/T_{P}$. This frequency should be high enough with respect to electrical resonances, as detailed in Equation (9). It should also be far enough from the mechanical amplification caused by the structural dynamics, which is commonly lower than the electrical resonant frequency, as surveyed by empirical estimates in [21]. It is worth noting that with this method, the physical complexity of implementing the exciters for the thin-film actuators can be significantly reduced. The value of *n* is determined by the output power of the electron gun, and $\vartheta_{i}$ can be determined through mechanics computation or experimental calibration. In practice, in case of the actuator saturation, an array of the electron guns might be optimally organized. In this study, although there are certainly more flexible methods for distributing power to the actuation nodes to morph the active reflector, we emphasize the principle with this simplest implementation.

### Macro-Scale Control

The control algorithm in this paper adopt the simple least squares (LS) reconstruction as studied extensively in [21,22,24], with a formulation of solving the input control efforts of overdetermined equations

(10) $$a={\left(J^{+}J\right)}^{-1}J^{T}s_{t}$$

where $s_{t}$ is the target shape and a represents the control efforts (e.g., voltages or flux energy in the quasi-static range). The matrix *J* is the interaction between the control inputs and the morphing outputs. This control can also be performed iteratively, preferably using damped least squares (DLS), which offers significant advantages in cases where there is a variation in *J* or when *J* is ill-conditioned [33].

**Figure 17:**
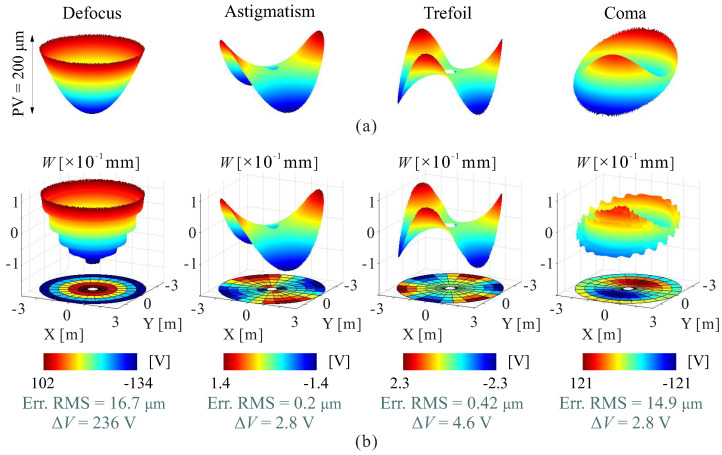
Numerical tests on evaluating the morphing capability of correcting primary optical errors of a surface. (**a**) target shapes of tested modes; (**b**) resulting shapes and voltage maps.

Figure 17 presents a numerical test evaluating the morphing capability of correcting primary optical errors of a surface. These errors are characterized by Zernike modes (as described in Appendix A), which are commonly used for optical surface examination. The results are consistent with those from [21,24], showing that morphing spherical mirrors face difficulties in forming the defocus and coma modes while achieving better results with the astigmatism and trefoil modes, both in terms of reduced residual errors and voltage consumption.

### Micro-Scale Control

Micro-scale control should be carried out after the macro-scale control, typically using the same algorithm as Equation (10). However, there are some differences that require slight modifications in solving the control efforts: (1) the actuator saturation is a concern due to the limited output power of the electron gun, and the applied potential must be positive along the polarization direction, for the driving energy $a_{i}$ in the vector a for the $i^{th}$ node, it should satisfy $0\leq a_{i}\leq E_{G}/n$, where $E_{G}$ is the overall output energy. In this study, the constrained LS problem is solved using a reflective Newton method, as described in [36]. (2) The matrix *J* contains significant uncertainties because the actuated area at this stage is much smaller than the entire structure, and the morphing behavior may be easily perturbed in terms of amplitudes. As discussed in Section 3.3, for this numerical test, we use a normalized amplitude Gaussian function (with $D_{S}=2P$) to form the interaction matrix *J* with a dimple morphing stroke of 1 μm; trigonometric or Bessel functions can also be considered for approximating the influence function, e.g., see [37].

**Figure 18:**
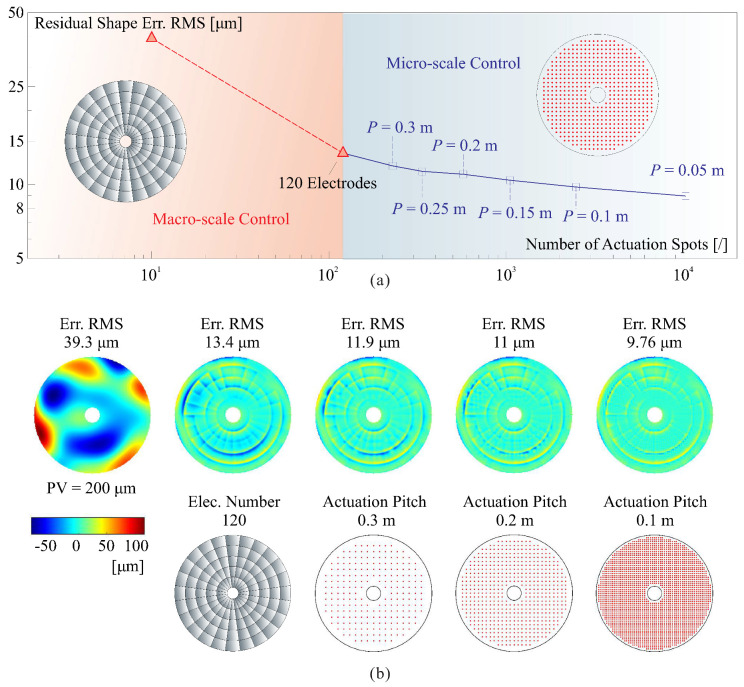
Correction of a random shape error using a hierarchical control approach that combines both macro- and micro-scale error correction. (**a**) Two-step correction reducing the surface error; the evaluation is promoted by using a small actuator pitch; (**b**) figures of surface errors for some cases.

Figure 18 demonstrates the correction of a random shape error using a hierarchical control approach that combines both macro- and micro-scale error corrections. In Figure 18a, the root mean square (RMS) residual error decreases with the number of actuators. The first step involves macro-scale control with an array of 120 electrodes, which corrects the original shape with an RMS error of $\sigma_{W}=39.3\;\mu$m to a roughly refined shape with an RMS error of $\sigma_{W}=13.4\;\mu$m. The print-through effect in the corrected shape can be observed in Figure 18b. The second step employs micro-scale control algorithms with various actuation node patterns, resulting in a gradual reduction of the residual shape error. It is worth noting that surface errors tend to be concentrated in the low spatial frequency components, so the reduction of error is significant in the first step. High-frequency errors can be gradually corrected by increasing the resolution of the actuation nodes in the second step. Figure 18b provides several examples of the figure error of the reflector shape.

## 5. Conclusions

This paper delves into the shape control of ultra-lightweight electro-actuated polymers with composite ferroelectric thin films. We commence with an overview of the development of thin-shell composites utilizing PVDF-TrFE film actuators. Our technology demonstrator, a 0.2 m diameter mirror with a 0.12 m clear pupil, marks a significant stride in precision optical and infrared systems. However, challenges loom when contemplating its application in larger systems spanning meters. Addressing constraints related to electrode size and managing numerous control channels is imperative for successful scalability. This necessitates achieving ultra-high-resolution strain actuation through non-contact charging, thereby reducing the number of input channels.

We investigate strain generation in thin-film actuators through both conventional electrode-based methods and non-contact electron flux excitation. The conventional approach involves a pair of parallel electrodes, leveraging piezoelectric and electrostrictive effects for strain generation. Our numerical studies incorporate experimentally calibrated ferroelectric material parameters. We delve into the film deposition process and potential limitations arising from the electrical unit. In contrast, we introduce an alternative non-contact actuation model employing electron flux. We provide a comprehensive analysis, including its equivalent circuit representation, potential distribution, and spatial effects on the thin film; a future experimental investigation is highly expected.

Furthermore, we present a vision for the future of large thin-shell reflectors by integrating the discussed methods for charging ferroelectric polymer films. While immediate implementation poses practical challenges, our research establishes the groundwork for potential applications in space observation and communication. We propose a hierarchical control strategy that combines macro-scale and micro-scale techniques, promising to rectify shape errors in thin-shell reflectors. We also offer a baseline design for power allocation in electron flux exciters, validated through numerical verification, streamlining thin-film actuator implementation. These strategies hold the potential to elevate precision and performance in future spaceborne observation systems, benefiting space exploration and communication technologies.

## Figures and Tables

**Table 1:** Experimentally measured ferroelectric parameters of the thin film, associated with Equation (2).

Deposition Technique	Spin Coating	Spray Coating		
Annealing condition	140 ${}^{\circ }$C	140 ${}^{\circ }$C	130 ${}^{\circ }$C	120 ${}^{\circ }$C
Relative permittivity $\varepsilon_{r}$ [/]	11.86	9.6	9.9	9.5
Piezoelectric constant $d_{31}$ [pC/N]	13.54	12.56	10.9	9.04
Electrostrictive constant $Q_{33}$ [m${}^{4}$/C${}^{2}$]	−12.65	−13.81	−12.27	−12.65
Remnant polarization $P_{S}$ [C/m${}^{2}$]	0.0152	0.0157	0.0148	0.0126

## Data Availability

Not applicable.

## Abbreviations

The following abbreviations are used in this manuscript:

AO	adaptive optics or active optics
CTE	coefficient of thermal expansion
DLS	damped least squares
DM	deformable mirror
DoF	degree-of-freedom
ESA	European Space Agency
ESO	European Southern Observatory
FoV	field of view
IAE	inflatable antenna experiment
JWST	James Webb Space Telescope
LS	least squares
MISSE	Materials International Space Station Experiment
ULB	Université Libre de Bruxelles
UV	Ultraviolet
PET	polyethylene terephthalate
PI	polyimide
PVDF	poly(vinylidene Fluoride
PVDF-HFP	poly(vinylidene fluoride-co-hexafluoropropylene)
PVDF-TrFE	poly(vinylidene fluoride-trifluoroethylene)
PWM	pulse width modulation
RMS	root mean square
VLT	very large telescope

## Appendix A

The Zernike mode $Z_{i}\left(\rho ,\theta \right)$ is defined on a unit circle in polar coordinates (ρ,θ), where 0<ρ<1, and it is usually used as a modal basis to describe the wavefront *w* on the pupil. Table A1 presents a list of analytical expressions of Zernike modes with an order of i=0−14 in the OSA/ANSI index, with a normalization of

(A1) $$\int_{0}^{2\pi }\int_{0}^{1}Z_{i}^{2}\rho d\rho d\theta =\pi$$

**Table A1:** Analytical expressions of the low-order Zernike modes (in OSA/ANSI’s index).

Term $Z_{n}^{m}$ (Index *i*)	Analytical Expression	Classical Name
$Z_{0}^{0}$ (0)	1	Piston
$Z_{1}^{-1}$ (1)	2ρsinθ	Vertical Tilt
$Z_{1}^{1}$ (2)	2ρcosθ	Horizontal Tilt
$Z_{2}^{-2}$ (3)	$\sqrt{6}\rho^{2}\sin2\theta$	Oblique Astigmatism
$Z_{2}^{0}$ (4)	$\sqrt{3}\left(2\rho^{2}-1\right)$	Defocus
$Z_{2}^{2}$ (5)	$\sqrt{6}\rho^{2}\cos2\theta$	Vertical Astigmatism
$Z_{3}^{-3}$ (6)	$\sqrt{8}\rho^{3}\sin3\theta$	Vertical Trefoil
$Z_{3}^{-1}$ (7)	$\sqrt{8}\left(3\rho^{3}-2\rho \right)\sin\theta$	Vertical Coma
$Z_{3}^{1}$ (8)	$\sqrt{8}\left(3\rho^{3}-2\rho \right)\cos\theta$	Horizontal Coma
$Z_{3}^{3}$ (9)	$\sqrt{8}\rho^{3}\cos3\theta$	Oblique Trefoil
$Z_{4}^{-4}$ (10)	$\sqrt{10}\rho^{4}\sin4\theta$	Oblique Quadrafoil
$Z_{4}^{-2}$ (11)	$\sqrt{10}\left(4\rho^{4}-3\rho^{2}\right)\cos2\theta$	Oblique Secondary Astigmatism
$Z_{4}^{0}$ (12)	$\sqrt{5}\left(6\rho^{4}-6\rho^{2}+1\right)$	Primary Spherical Aberration
$Z_{4}^{2}$ (13)	$\sqrt{10}\left(4\rho^{4}-3\rho^{2}\right)\cos2\theta$	Vertical Secondary Astigmatism
$Z_{4}^{4}$ (14)	$\sqrt{10}\rho^{4}\cos4\theta$	Vertical Quadrafoil
